# 68415 Neural Impact of Neighborhood Disadvantage in Traumatically-Injured Adults: a Multi-Modal Investigation

**DOI:** 10.1017/cts.2021.462

**Published:** 2021-03-30

**Authors:** E. Kate Webb, Carissa Weis, Ken Bennett, Ashley Huggins, Elizabeth Parisi, Jessica Krukowski, Terri deRoon-Cassini, Christine Larson

**Affiliations:** 1 University of Wisconsin-Milwaukee; 2 Montana VA Healthcare System; 3 Marquette University; 4 Medical College of Wisconsin

## Abstract

ABSTRACT IMPACT: Neighborhood disadvantage was significantly associated with brain structure and function in trauma-exposed adults, providing evidence that contextual factors should be assessed in mental health research, particularly in high-risk populations. OBJECTIVES/GOALS: Over 13 percent of Americans live in a socioeconomically disadvantaged neighborhood. Previous work has linked lower individual socioeconomic position to alterations in brain structure and function. However, the neural effects of area-level socioeconomic factors, such as neighborhood disadvantage, are unclear. METHODS/STUDY POPULATION: We recruited two-hundred and fifteen traumatically-injured participants from an Emergency Department in southeastern Wisconsin. An Area Deprivation Index (ADI) score, a national measure of neighborhood socioeconomic disadvantage, was derived from each participant’s home address. Two-weeks post-trauma, participants underwent a battery of self-report measures and functional magnetic resonance imaging (fMRI) scans. Using a multi-modal approach, we investigated the impact of ADI on brain structure as well as neural activation during rest and during an emotional uncertainty task. We sought to disentangle the relationship between neighborhood and individual socioeconomic position and neural activity in the context of trauma. RESULTS/ANTICIPATED RESULTS: We demonstrated that neighborhood disadvantage is associated with decreased volume and alterations of resting state functional connectivity of structures implicated in affect processing, including the hippocampus, amygdala, and ventromedial prefrontal cortex. These results held even after controlling for relevant individual variables, including acute post-traumatic stress symptoms and years of education. Moreover, individuals from disadvantaged neighborhoods exhibited heighted activation of these same structures in response to aversive stimuli. Thus, brain regions critical for recognizing and processing negative stimuli are susceptible to the effects of area-level socioeconomic factors. DISCUSSION/SIGNIFICANCE OF FINDINGS: The results offer additional evidence that neurobiological mechanisms clarify how stress ‘gets under the skin’. Changes to key brain regions may explain why those living in disadvantaged neighborhoods are at a heighted risk of PTSD. Broadly, these findings should inform future policies and community-driven interventions aimed at reducing poverty.

